# Fructans redistribution prior to sprouting in stored onion bulbs is a potential marker for dormancy break

**DOI:** 10.1016/j.postharvbio.2018.12.002

**Published:** 2019-03

**Authors:** I.C. Ohanenye, M.C. Alamar, A.J Thompson, L.A. Terry

**Affiliations:** Plant Science Laboratory, Cranfield University, Bedfordshire, MK43 0AL, UK

**Keywords:** *Allium cepa*, Sprouting, Deficit irrigation, Fructans

## Abstract

Continuous supply of high quality onion bulbs to meet year-round demand is dependent on maintaining dormancy and bulb quality during storage. Sprouting impacts negatively on the storage quality of onion bulbs. Ethylene supplementation has previously been revealed to inhibit sprout growth in stored onion bulbs. Fructans content, especially those at higher degree of polymerisation (DP), are reported to positively correlate with delayed sprouting. However, little is known about the impact of pre-harvest irrigation regimes on fructans accumulation and redistribution in relation to onion bulb dormancy and quality in store. Across two seasons, onion plants of cultivars ‘Red Baron’ and ‘Sherpa’ were subjected to full irrigation (FI) (100% replenishment of crop evapotranspiration) or deficit irrigation (DI) (50% of FI treatment) from bulb initiation to harvest. Bulbs were harvested at full maturity and stored at 1 °C for five months. Bulbs were treated with or without 1-MCP (1 μL L^−1^) for 24 h before storage under continuous ethylene supplementation (10 μL L^−1^) or air. DI had no effect on dormancy-break, sprout emergence, total fructans content and total sugar content. In contrast, ethylene delayed sprout emergence and suppressed sprout growth; added 1-MCP enhanced this effect. The concentration of DP3-8 fructans were higher in top and bottom sections compared to the baseplate. Before sprout emergence, fructans of DPs 7–8 were no longer present in the top and bottom wedges, while they accumulated in the baseplate; irrespective of pre- or postharvest treatments. This redistribution of fructans within the bulb suggested a transition in dormancy state and could be used as a predictive marker for sprouting in stored onion bulbs.

## Highlights

• Deficit irrigation neither influenced sprouting nor the accumulation of fructans.
• Top-to-bottom redistribution of fructans occurred in onion bulb prior to sprouting.
• Fructans redistribution could be a predictive marker for onion dormancy-break.
• Ethylene and 1-MCP may regulate eco-dormancy; but not endo-dormancy.

## 1 Introduction

Despite being a seasonal crop, the demand for onion bulbs is all-year-round, therefore, the onion industry relies on innate dormancy and storage treatments to extend availability. Dormancy break elicits various changes including increased weight-loss, sprouting and breakdown of sugars and fructans. Currently, the industrial standard sprout suppressant regime involves the use of maleic hydrazide (MH) - applied as a foliar spray - in conjunction with low temperature storage and, occasionally controlled atmosphere (CA).

Ethylene supplementation has previously been shown to delay and suppress sprouting in stored onion bulbs. Onion bulbs are low endogenous ethylene producers (Cools et al., 2011; Chope et al., 2012); nonetheless, continuous exogenous ethylene supplementation at 10 μL L^−1^ during storage suppresses sprout growth (Briddon and Sbeu, 2006; Bufler, 2009; Cools et al., 2011; Chope et al., 2012). Further evidence of ethylene sprout suppression in other crops was revealed by Foukaraki et al. (2014) (potatoes) and Amoah et al. (2016) (sweet potatoes). Perhaps unexpectedly, the treatment of onion bulbs with 1-methylcyclopropene (1-MCP) - a known inhibitor of ethylene activities – also suppressed sprout growth and caused the accumulation of sugars (Chope et al., 2006a); and this sprout suppression was further enhanced when applied in combination with ethylene supplementation (Cools et al., 2011).

Fructans are polymers of fructose and the major reserve carbohydrates in onion bulbs, which vary in their degree of polymerisation (DP) (Valluru and Van Den Ende, 2008). Fructans and simple sugars are known to decrease in bulbs during dormancy transition (Chope et al., 2012). The concentration of fructans at harvest was reported to positively correlate with long storage and delayed sprouting in onion bulbs (Suzuki and Cutcliffe, 1989; Jaime et al., 2001; Benkeblia et al., 2006). Furthermore, Jaime et al. (2001), reported a positive correlation between long storing onion bulbs and higher DP fructans content compared to poorer storing onion bulbs, yet, the mechanism of fructans mobilisation and metabolism in relation to onion dormancy, is still unclear.

Previous studies on the role of deficit irrigation (DI) on the postharvest qualities of onion bulbs are contradictory. Rattin et al. (2011) and Vickers et al. (2015) reported that DI caused early sprouting and greater postharvest losses; Leskovar et al. (2012) reported an increase in postharvest storage life; while Martín de Santa Olalla et al. (2004) and Enciso et al. (2009) found no such differences. Notably, none of these earlier studies combined DI with any postharvest sprout mitigation treatments. Moreover, DI had previously been shown to cause the accumulation of non-structural carbohydrates such as sugars in strawberry (Bordonaba and Terry, 2010) and tomato fruits (Kirda et al., 2004). In *Aloe vera* leaves, DI treatments caused up to 60% increase in the fructans content when compared to the fully irrigated plants (Salinas et al., 2016). In the leaves of transgenic tobacco, Pilon-Smits et al., 1995a, Pilon-Smits et al., 1995b reported that polyethylene glycol-mediated drought stress caused a 7-fold increase in fructans concentration and overall biomass, while starch level was not affected. Similarly, drought stress caused the accumulation of fructans in the shoots and roots of transgenic sugar beets (Pilon-Smits et al., 1999) and in the rhizophores (the storage organ) of *Vernonia herbacea* (Garcia et al., 2011). Salinas et al. (2016) further reported that DI caused the accumulation of fructans with higher DP when compared to fully irrigated leaves of *Aloe ver*a plants. Despite these reports, and the implication that fructans may regulate onion dormancy (Suzuki and Cutcliffe, 1989; Jaime et al., 2001; Benkeblia et al., 2006; Chope et al., 2012), it remains unknown if fructans content can be manipulated through DI.

The aim of this study was to investigate the influences of pre-harvest DI and postharvest ethylene supplementation on the accumulation and distribution of fructans in relation to dormancy-break and sprouting in stored onion bulbs.

## 2 Materials and methods

### 2.1 Plant material

In this study, two different onion cultivars (‘Red Baron’ and ‘Sherpa’) were used in 2015 and only ‘Sherpa’ in 2016. In 2015, ‘Red Baron’ sets and ‘Sherpa’ seeds were sourced from Elsom Seeds (Lincs., UK), and Steve Howe Seeds (Lincs., UK), respectively; while in 2016, ‘Sherpa’ seeds were sourced from Limagrain (Lincs., UK). For both years, ‘Sherpa’ seeds were planted into trays with John Innes No 1 seed media and seedlings were transplanted into pots six weeks after germination, while ‘Red Baron’ were planted directly into pots. Final growth media was John Innes Compost No 3. Equal weights of the growth media (7.3 kg) were measured into 96 pots of 8 L capacity per cultivar for 2015 and 264 pots for 2016 experiments. Plants were transplanted at the rate of three plants per pot (used as pseudo-reps) for both years. Plants were split into three completely randomised blocks (replicates) formed across two benches in the glasshouse. The first and last rows of plants in blocks 1 and 3, respectively, served as guard plants (not included in the analysis) for both years. Plants were fertilised with 800 mL of Hoagland’s solution as two single 400 mL applications per treatment. For both years, bulbs were harvested manually at full maturity when all plant foliage had lodged (100% fall-down) and were cured under glass for six weeks (August - September).

### 2.2 Experimental design

Plants were subjected to two pre-harvest treatments: full irrigation (FI) and deficit irrigation (DI); where FI amounted to 100% replenishment of crop evapotranspiration (ET_c_), and DI corresponded to 50% of the FI treatment. Irrigation was uniformly applied using an automated irrigation system (AC4, Hozelock, Warwickshire, UK). Polyvinyl tubing (20 mm diameter) were fitted with pressure-compensating emitters of 1.2 L min^−1^ flow rate, which were connected to polyvinyl tubes (5 mm diameter) to the pots (one emitter per pot). Soil moisture content was monitored weekly both gravimetrically (based on individual pot weight) and volumetrically using soil moisture probes (HH2 and ML2x, Delta-T, Cambs., UK); three pots per treatment per block were assessed. The onion plants were subjected to differential pre-harvest irrigation treatments for seven weeks - from bulb initiation stage until two weeks before harvest, when no more irrigation was applied thereafter.

At harvest, all three bulbs per pot were collected, tagged and weighed together. Afterwards, the bulbs were spread out in a single layer on the benches in the glasshouse for curing (18–35 °C and 40–90 % relative humidity). Bulbs were weighed weekly throughout the six weeks’ curing period. After curing, bulbs were transferred to 100 L storage boxes and stored at 1 °C for 18 and 20 weeks for 2015 and 2016, respectively. For 2015, bulbs were subjected to two treatments: continuous ethylene supplementation at 10 μL L^−1^ or continuous air, as described elsewhere (Chope et al., 2006a; Amoah et al., 2017). For 2016, bulbs were treated with or without 1-MCP at 1 μL L^−1^ for 24 h before storage; and then stored in air or under continuous ethylene supplementation at 10 μL L^−1^ (Cools et al., 2011). Thus, the postharvest treatments were: air, air + ethylene, 1-MCP + air, and 1-MCP + ethylene. At each sampling point, three bulbs were collected in triplicate, per treatment and throughout the storage duration (3 bulbs x 3 replicates x treatments). All experiments (pre- and postharvest) were arranged in a completely randomised design.

Bulbs were stored for 18 weeks in 2015 with six postharvest sampling points, while in 2016, bulbs were stored for 20 weeks with seven postharvest sampling points with two pre-storage sampling points (pre-harvest and mid-curing) added. The pre-harvest samples for 2016 were collected at the termination of irrigation at 100% fall-down (two weeks before harvest), while the mid-curing samples were collected three weeks into curing. Time 0 (week 0) samples for both years were collected at the end of curing, prior to bulbs being transferred to the cold room. Afterwards, sampling was conducted bi-weekly until sprout length was 40% in proportion to bulb height (Chope, 2006). At each sampling point, bulb weight, real-time respiration rate and sprout assessment (sprout length as a percentage of total bulb height) were recorded. Bulbs were sliced in two equal parts from top to bottom with a sharp stainless-steel knife; then, the individual baseplates were excised, after which the remaining section (including the skin) was divided equally into top and bottom (Supplement 6). All samples were immediately snap-frozen in liquid nitrogen and stored at −80 °C before lyophilisation at -55 °C in a freeze-drier (Scanvac, Lynge, Denmark) in the dark for 7 days. Freeze-dried samples were stored at −40 °C prior to biochemical analyses.

### 2.3 Estimation of crop evapotranspiration (ET_c_) and soil moisture measurements

Three pots per block, per treatment, per cultivar were randomly selected, weighed and reweighed after 24 h. The ET_c_ was then calculated as the difference between the recorded initial and final weights (in grams). Soil moisture content were measured using Delta-T soil moisture probes (type: HH2 and ML2x, Delta-T, Cambs., UK). The probes were inserted fully into the soil and the percentage soil moisture content reading was recorded. Measurements were conducted weekly, from bulb initiation to harvest (June – August) for both years. Where there was a need, irrigation was readjusted according to the treatment.

### 2.4 Curing and storage bulb weight-loss

Onion bulb weight-loss was measured weekly during curing and at each sampling point during storage.

### 2.5 Real-time respiration rate (RR)

Real-time respiration rate measurements were taken at each sampling point using the Sable Respirometry System (Model 1.3.8 Pro, Sable Systems International, NV, USA), as described previously by Collings et al. (2013). Each replicate (3 replicates x 3 onion bulbs per replicate) were taken out from the storage boxes and placed on the laboratory bench for a minimum of one hour to be acclimatised to room temperature. Each replicate was then placed in a 3 L sealed gas jar with gas inlet for air supply and outlet for respiration rate measurement as CO_2_ production. The measured CO_2_ produced (in mL h^−1^) was adjusted to get the final respiration rate values as CO_2_ produced in milligrams per kilogram of bulb weight per hour (mg kg^−1^ h^−1^) as previously described by Collings et al. (2018).

### 2.6 Sprout assessment

Sprout incidence were assessed as previously described elsewhere (Chope et al., 2006a, 2006b). Onion bulbs were cut in half (vertically from top to bottom) and internal sprout length was presented as a percentage of the total bulb height (Appendix A).

### 2.7 Non-structural carbohydrates extraction and quantification

Freeze-dried samples (top wedge, bottom wedge and baseplate [Appendix B]) were ground into fine powder using metallic ball bearings (size: 5 mm) placed in each tube with freeze-dried samples and placed into a Star-beater (VWR International bvba, Leuven, Belgium). Non-structural carbohydrates (NSCs) from the powdered samples were extracted and quantified as described elsewhere, with modifications. Briefly, 150 mg of pulverised samples were thoroughly vortexed in 3 ml of 62.5% (v/v) aqueous HPLC grade methanol, incubated in a shaking bath for 15 min. at 55 °C and filtered through a 13 mm diameter × 0.2 μm PTFE filter unit (Jaytee Biosciences Ltd, Kent, UK). Extracts (10 μL) were injected into a Prevail Carbohydrate ES (250 × 4.6 mm, 5 μm particle size; Grace Discovery Sciences, IL, USA) with a guard column (ZORBAX Eclipse Plus-C18, 2.1 × 12.5 mm, 5 μm particle size) in Agilent 1260 Infinity HPLC system coupled to an Evaporative Light-Scattering Detector (Agilent Technologies LDA UK Limited, Cheshire, UK). The mobile phase was a linear increase/decrease amount of water in acetonitrile (% water): 0–15 minutes, 20–50 %; 15–20 minutes, 50-20%; 20–23 minutes, 20-15%; 23–30 minutes, 15-10%. The flow rate and injection volumes were 0.8 mL min^−1^ and 5 μL, respectively, and the oven temperature was set at 30 °C. The presence and concentration of fructose, glucose, sucrose, kestose and nystose were calculated by comparing the peak areas in each sample to standards (0.05–5 mg mL^−1^) using Agilent ChemStation software version 4 (Agilent Technologies, CA, USA). Fructose, glucose, sucrose, kestose and nystose standards were obtained from Sigma-Aldrich Co. (Dorset, UK). Fructans with degree of polymerisation higher than four were quantified using the standard curve for nystose as previously described by Downes (2010). Total sugar content was calculated as the sum of the concentrations of fructose glucose and sucrose while total fructans was calculated as the sum of the concentrations of fructans of DP3-8 on a per section and per sample bases.

### 2.8 Statistical analyses and plots

All statistical analyses were conducted using Genstat for Windows 10th Edition (VSN International Ltd, Herts., UK). Analysis of variance (ANOVA) was performed to identify factors that significantly affected variance in the physiological and biochemical data collected. ANOVA was performed on the data specifying a nested treatment structure of a common baseline (observation before postharvest treatments). Least significant difference (LSD) values were calculated from each analysis, for comparison of appropriate treatment means, using general analysis of variance. A significance threshold of p < 0.05 was adopted, for all analyses. SigmaPlot for Windows SPW13 (Systat Software, Inc., London, UK) was used for all plots.

## 3 Results

### 3.1 Crop evapotranspiration, soil moisture content and curing weight-loss

Mean crop evapotranspiration (ET_c_), soil moisture content and curing weight-loss (Appendix C– Fig. B1[2015] and B [2016]) varied between pre-harvest treatments. A comparison between cultivars showed that curing weight-loss was 15.0 and 8.6% for ‘Red Baron’ FI and DI bulbs, respectively, compared to 9.8 and 6.2% for ‘Sherpa’ FI and DI bulbs, respectively, in 2015. While a comparison between years showed that curing weight-loss for ‘Sherpa’ in 2015 was 9.8 and 6.2% for FI and DI bulbs, respectively, compared to 6.8 and 3.6% for FI and DI bulbs, respectively, in 2016 (Appendix C – Fig. B1). Interestingly, weight-loss during curing was twice as high in FI compared to DI, irrespective of cultivar or year. Bulb storage weight-loss was continuous throughout the storage period, however, there were no significant differences between pre- or postharvest treatments (Appendix C – Fig. B1 [2015] and D [2016]). Overall, bulb weight was reduced in DI bulbs for all years; such that in 2015, bulb weights were significantly (12.2 and 41.8%) higher for fully irrigated ‘Red Baron’ and ‘Sherpa’ onion bulbs compared to the DI bulbs, respectively. For 2016, ‘Sherpa’ bulb weights were significantly (14%) higher for FI compared to DI bulbs.

### 3.2 Sprout length

**Fig. 1:**
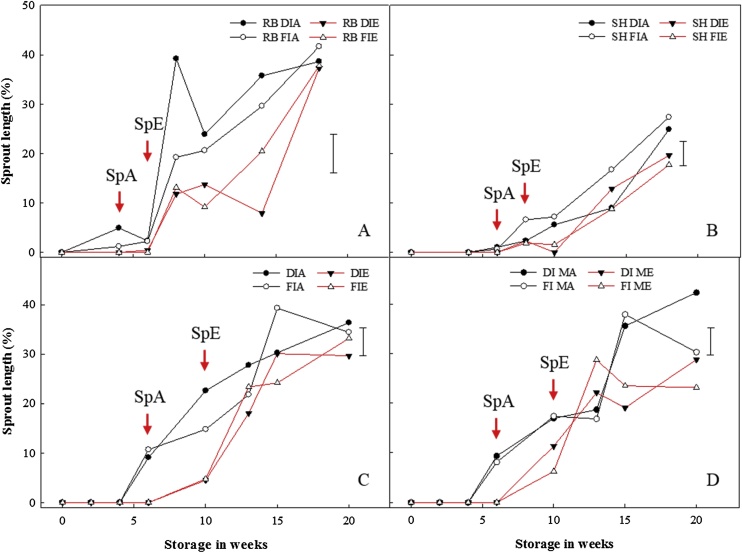
Sprout length in stored onion bulbs. Sprout length was measured as sprout length in proportion to bulb height represented as a percentage for onion bulbs of cultivar ‘Red Baron’ (RB) and ‘Sherpa’ (SH) grown in 2015 (A and B) and 2016 (C and D) under full irrigation (FI) or deficit irrigation (DI); where FI amounted to 100% replenishment of crop evapotranspiration (ET_c_) and DI was ET_c_ replenishment at 50%. Bulbs were harvested at full maturity (100% fall-down) and cured under glass for six weeks for both years. After curing, bulbs were either treated with 1-MCP at 1 μL L^−1^ for 24 h or untreated before storage; and then, stored at 1 °C under continuous ethylene supplementation at 10 μL L^-1^ or air. DIA and FIA are DI and FI bulbs in air, DIE and FIE are DI and FI bulbs stored under ethylene, DIMA and FIMA are DI and FI bulbs treated with 1-MCP and stored in air and DIME and FIME are DI and FI bulbs treated with 1-MCP and stored under ethylene. SpA and SpE are sprout emergences for bulbs stored in air and ethylene, respectively. LSD bar at 95% confidence shown.

There was no significant difference in sprout emergence between pre-harvest treatments, irrespective of cultivar or year (Fig. 1). However, sprout emergence and sprout length varied according to the postharvest regime. Ethylene delayed sprout emergence by two and four weeks (2015 and 2016, respectively) and suppressed sprout growth compared to bulbs stored in air. Overall, 1-MCP-treated bulbs stored under ethylene produced the shortest sprouts followed by untreated bulbs under ethylene supplementation, untreated bulbs stored in air and 1-MCP treated bulbs stored in air.

### 3.3 Fructans content

**Fig. 2:**
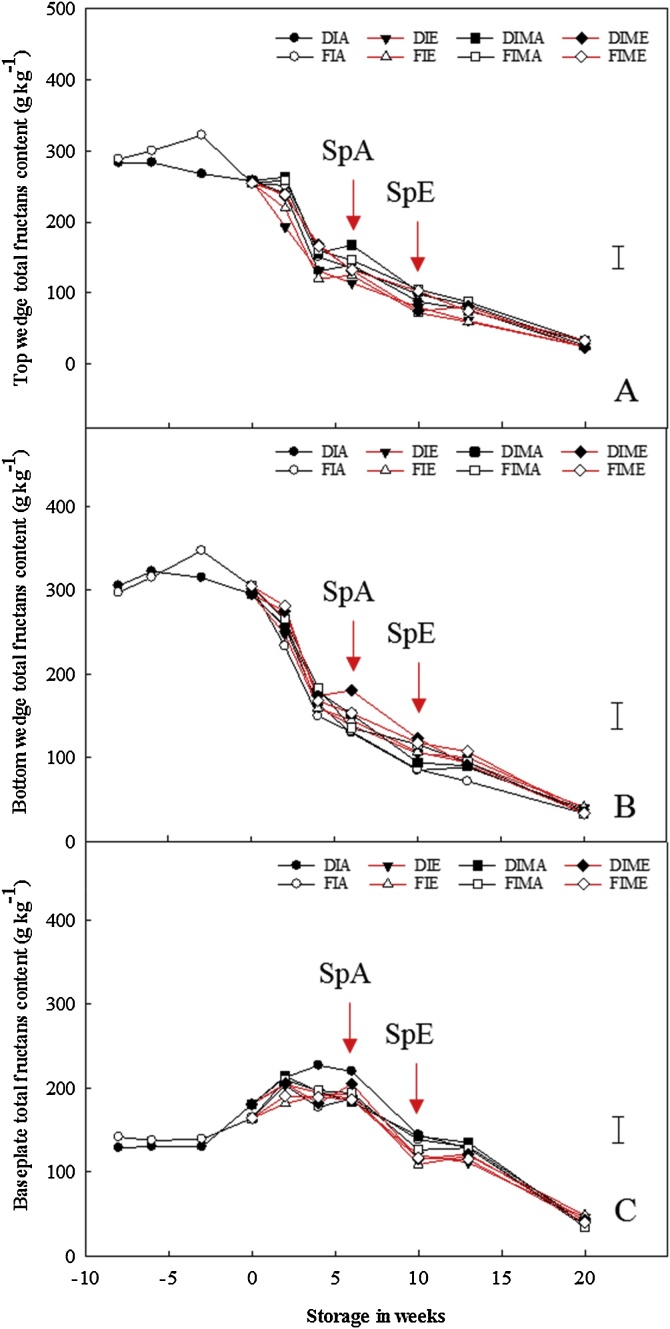
Pre- and postharvest total fructans content in the top wedge, bottom wedge and baseplate of stored onion bulbs treated or untreated with 1-MCP. Total fructans contents (sum of fructans of DP3-8 content) per dry weight for the top wedge (A), bottom wedge (B) and baseplate (C) of onion bulbs of cultivar ‘Sherpa’ grown in 2016 under full irrigation (FI) or deficit irrigation (DI); where FI amounted to 100% replenishment of crop evapotranspiration (ET_c_) and DI was ET_c_ replenishment at 50%. Bulbs were harvested at full maturity (100% fall-down) and cured under glass for six weeks for both years. Post-curing, bulbs were treated with 1-MCP at 1 μL L^−1^ for 24 h before storage or untreated with 1-MCP, and were stored at 1 °C under continuous ethylene supplementation at 10 μL L^-1^ or air. DIMA and FIMA are DI and FI bulbs treated with 1-MCP and stored in air and DIME and FIME are DI and FI bulbs treated with 1-MCP and stored under ethylene. SpA and SpE are sprout emergences for bulbs stored in air and ethylene, respectively. Where pre-harvest = –8; harvest = –6; mid-curing = –3; and end of curing = 0 in weeks. LSD bar at 95% confidence shown.

Neither pre- nor postharvest treatments significantly influenced the total fructans content within the bulbs (Fig. 2). The highest total fructans content (sum of DP3-8 fructans concentration) in the top (283–288.6 g kg^−1^) and bottom (297–305.3 g kg^−1^) sections were measured at pre-harvest and this declined continuously throughout the storage period; irrespective of pre- or postharvest treatments. In contrast, the total fructans content for the baseplate (128–141.7 g kg^−1^) increased continuously, irrespective of ethylene treatment, from pre-harvest until six weeks of storage (at sprout emergence for bulbs stored in air), before gradually declining continuously thereafter. Nevertheless, there were no significant differences between pre-harvest treatments as independent factors (p = 0.365), or with ethylene (p = 0.404) and 1-MCP (p = 0.627).

**Fig. 3:**
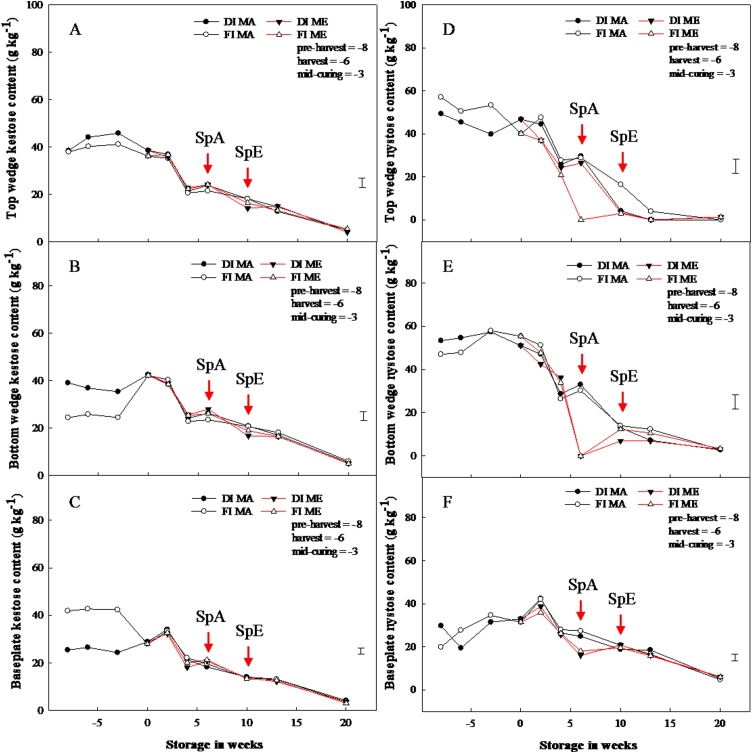
Pre- and postharvest kestose and nystose contents in the top section, bottom section and baseplate of stored onion bulbs treated with and without 1-MCP and ethylene. Kestose and nystose contents per dry weight for the top wedge (A and D), bottom wedge (B and E) and baseplate (C and F) of onion bulbs of cultivar ‘Sherpa’ grown in 2016 under full irrigation (FI) or deficit irrigation (DI); where FI amounted to 100% replenishment of crop evapotranspiration (ET_c_) and DI was ET_c_ replenishment at 50%. Bulbs were harvested at full maturity (100% fall-down) and cured under glass for six weeks for both years. Post-curing, bulbs were treated with 1-MCP at 1 μL L^−1^ for 24 h before storage, and were stored at 1 °C under continuous ethylene supplementation at 10 μL L^-1^ or air. DIMA and FIMA are DI and FI bulbs treated with 1-MCP and stored in air and DIME and FIME are DI and FI bulbs treated with 1-MCP and stored under ethylene. SpA and SpE are sprout emergences for bulbs stored in air and ethylene, respectively. LSD bar at 95% confidence shown.

The kestose (DP3) content between harvest and mid-curing in the bottom wedge was significantly higher in DI compared to FI, while for the baseplate, DP3 was significantly higher in FI compared to DI bulbs. However, at the end of curing, these differences had disappeared. No similar differences were found for nystose (Fig. 3) or other fructans of higher DP; irrespective of pre-harvest treatments or year. Moreover, by the end of curing and during storage, the differences in the accumulation of kestose between pre-harvest treatments had ceased to exist. Besides a significant decline in the nystose content for the bottom section and baseplates of 1-MCP-treated bulbs stored under ethylene at week 6, there was no consistent effect of ethylene or 1-MCP on kestose or nystose contents during storage.

**Fig. 4:**
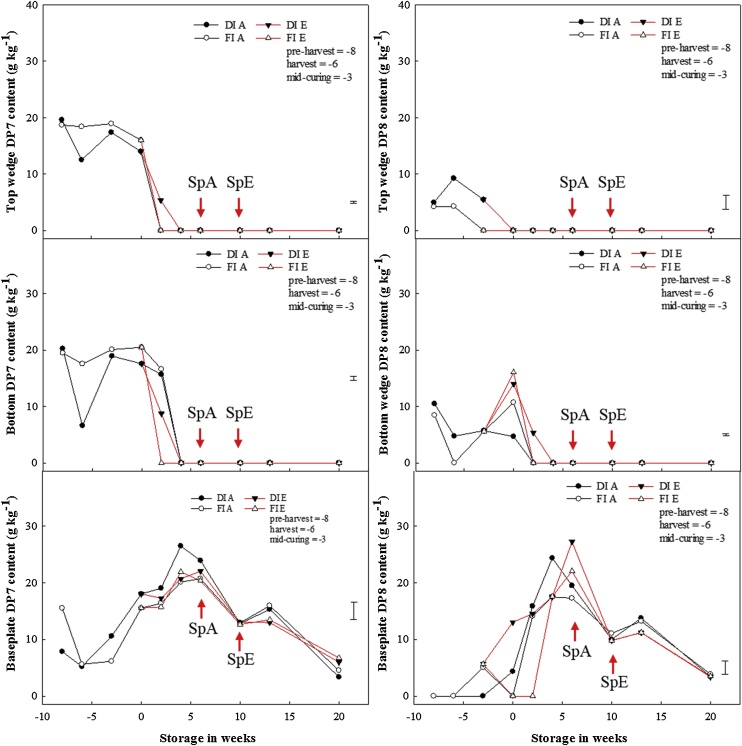
Pre- and postharvest fructans of DP7 and 8 contents for the top section, bottom section and baseplate of stored onion bulbs. Fructans of DP7 and 8 contents per dry weight for the top wedge, bottom wedge and baseplate of onion bulbs of cultivar ‘Sherpa’ grown in 2016 under full irrigation (FI) or deficit irrigation (DI); where FI amounted to 100% replenishment of crop evapotranspiration (ET_c_) and DI was ET_c_ replenishment at 50%. Bulbs were harvested at full maturity (100% fall-down) and cured under glass for six weeks for both years. Post-curing, bulbs were stored at 1 °C under continuous ethylene supplementation at 10 μL L^−1^ or air. DIA and FIA are DI and FI bulbs stored in air and DIE and FIE are DI and FI bulbs stored under ethylene. SpA and SpE are sprout emergences for bulbs stored in air and ethylene, respectively. LSD bar at 95% confidence shown.

For 2015 onion bulbs, fructans of DP3-6 were present across all sections in the cured bulbs (DP6 shown in Appendix D); while fructans of DP7 and 8 were present only in the baseplate. For 2016 onion bulbs, fructans of DP3-7 were present across all sections at pre-harvest, harvest, and mid-curing stages, with DP8 fructans being totally absent in the baseplate. However, at the end of curing, irrespective of pre-harvest treatments, DP8 fructans were no longer present in the top wedge (Fig. 4).

Prior to sprout emergence, there was a significant decline in fructans content, especially for fructans of DP6 and above. For 2015 bulbs, fructans of DP6 in the top and bottom wedges declined from *ca*. 47.7 to 0 g kg^−1^ (Appendix D). A similar decline was found in 2016 bulbs. Fructans of DPs 7 and 8 (Fig. 4) in the top and bottom wedges declined from 20.5 to 0 and 16 to 0 g kg^−1^ DW, for DPs 7 and 8, respectively. In contrast to the decline of higher DP fructans in the top and bottom wedges, they increased in the baseplate; irrespective of cultivar, pre- or post-harvest treatments or year.

Correlations were found to be stronger between fructans of closer DPs (e.g. DP3 and DP4) when compared to fructans with DPs wider apart (e.g. DP3 and DP8) as shown in Table 1.

**Table 1:** Correlation between sugars, fructans, total sugars, total fructans and total non-structural carbohydrates for onion bulbs grown under deficit irrigation, treated with 1-methylcyclopropene (at 1 μL L^−1^) 24 h prior to storage and stored under continuous ethylene (at 10 μL L^−1^) or air.

Compounds	Fructose	Glucose	Sucrose	DP3	DP4	DP5	DP6	DP7	DP8	Total Sugars	Total fructans	Total NSc
Fructose	1.00	0.81	0.66	0.44	0.07	−0.05	−0.19	−0.33	−0.33	0.90	0.12	0.68
Glucose	0.81	1.00	0.77	0.77	0.51	0.37	0.17	−0.08	−0.28	0.96	0.53	0.91
Sucrose	0.66	0.77	1.00	0.83	0.60	0.54	0.39	0.20	0.05	0.87	0.68	0.92
DP3	0.44	0.77	0.83	1.00	0.83	0.77	0.58	0.31	0.02	0.75	0.89	0.93
DP4	0.07	0.51	0.60	0.83	1.00	0.88	0.73	0.58	0.25	0.43	0.95	0.73
DP5	−0.05	0.37	0.54	0.77	0.88	1.00	0.86	0.70	0.41	0.31	0.95	0.64
DP6	−0.19	0.17	0.39	0.58	0.73	0.86	1.00	0.81	0.57	0.13	0.84	0.47
DP7	−0.33	−0.08	0.20	0.31	0.58	0.70	0.81	1.00	0.71	−0.09	0.66	0.23
DP8	−0.33	−0.28	0.05	0.02	0.25	0.41	0.57	0.71	1.00	−0.22	0.35	0.00
Total Sugars	0.90	0.96	0.87	0.75	0.43	0.31	0.13	−0.09	−0.22	1.00	0.48	0.92
Total fructans	0.12	0.53	0.68	0.89	0.95	0.95	0.84	0.66	0.35	0.48	1.00	0.79
Total NSC	0.68	0.91	0.92	0.93	0.73	0.64	0.47	0.23	0.00	0.92	0.79	1.00

### 3.4 Total sugar (fructose, glucose, and sucrose) content

**Fig. 5:**
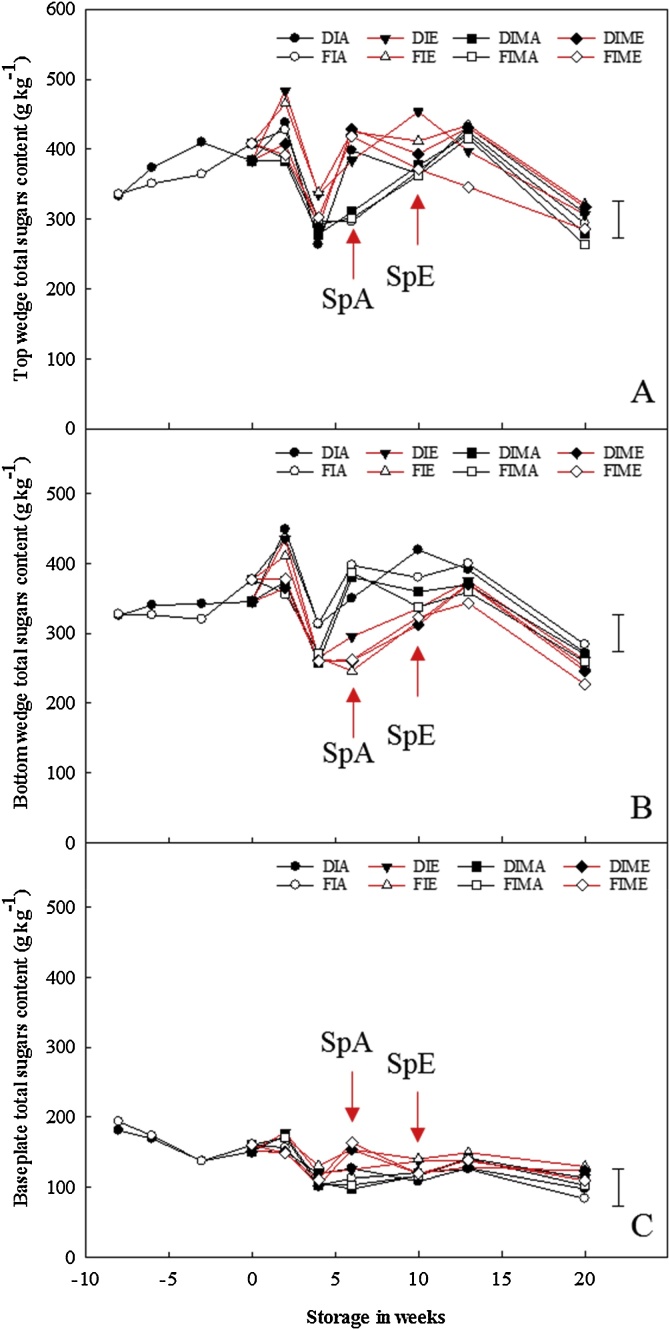
Pre- and postharvest total sugar content in the top section, bottom section and baseplate of stored onion bulbs treated with and without 1-MCP and ethylene. Total fructans contents (sum of fructose glucose and sucrose content) per dry weight for the top wedge (A), bottom wedge (B) and baseplate (C) of onion bulbs of cultivar ‘Sherpa’ grown in 2016 under full irrigation (FI) or deficit irrigation (DI); where FI amounted to 100% replenishment of crop evapotranspiration (ET_c_) and DI was ET_c_ replenishment at 50%. Bulbs were harvested at full maturity (100% fall-down) and cured under glass for six weeks for both years. Post-curing, bulbs were treated with 1-MCP at 1 μL L^−1^ for 24 h before storage or untreated with 1-MCP, and were stored at 1 °C under continuous ethylene supplementation at 10 μL L^-1^ or air. DIMA and FIMA are DI and FI bulbs treated with 1-MCP and stored in air and DIME and FIME are DI and FI bulbs treated with 1-MCP and stored under ethylene. SpA and SpE are sprout emergences for bulbs stored in air and ethylene, respectively. Where pre-harvest = –8; harvest = –6; mid-curing = –3; and end of curing = 0 in weeks. LSD bar at 95% confidence shown.

There were no significant differences in the total sugar content (the sum of fructose, glucose, and sucrose concentrations) between deficit and fully irrigated bulbs. Moreover, the total sugar content declined across all sections after four weeks of storage, with no significant differences between pre- and postharvest treatments (Fig. 5); a similar trend was found in 2015.

**Fig. 6:**
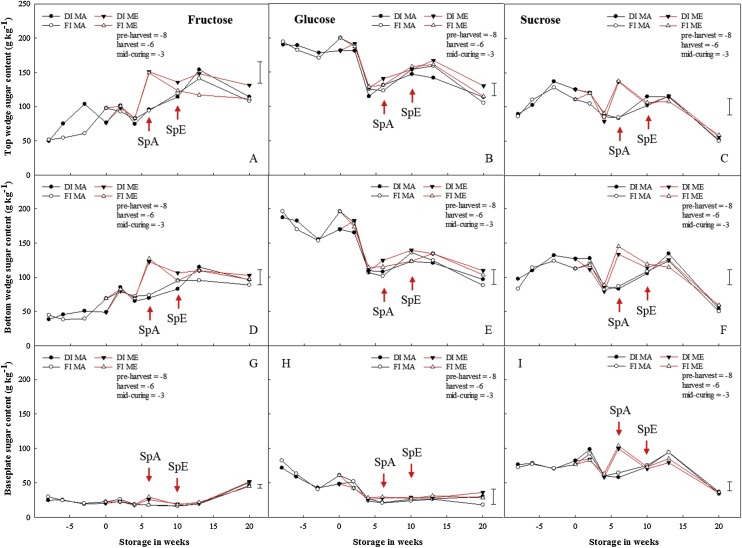
Pre- and postharvest sugar content in stored onion bulbs treated with and without 1-MCP and ethylene. Fructose, glucose and sucrose contents per dry weight for the top wedge (A, B and C), bottom wedge (D, E and F) and baseplate (G, H and I) of onion bulbs of cultivar ‘Sherpa’ grown in 2016 under full irrigation (FI) or deficit irrigation (DI); where FI amounted to 100% replenishment of crop evapotranspiration (ET_c_) and DI was ET_c_ replenishment at 50%. Bulbs were harvested at full maturity (100% fall-down) and cured under glass for six weeks for both years. After curing, bulbs were treated with 1-MCP at 1 μL L^−1^ for 24 h before storage and were stored at 1 °C under continuous ethylene supplementation at 10 μL L^-1^ or air. DIMA and FIMA are DI and FI bulbs treated with 1-MCP and stored in air and DIME and FIME are DI and FI bulbs treated with 1-MCP and stored under ethylene. SpA and SpE are sprout emergences for bulbs stored in air and ethylene, respectively. LSD bar at 95% confidence shown.

Overall, fructose content increased continuously throughout the storage period. There were no significant differences in the fructose and sucrose contents for 1-MCP untreated bulbs stored under ethylene. In contrast, fructose and sucrose contents increased by 1.5-fold for 1-MCP treated bulbs stored under ethylene (Fig. 6). Glucose and sucrose contents increased slightly in the first two weeks of storage before they declined by a half across all sections after four weeks of storage (prior to sprout emergence); nevertheless, there were no differences between pre-harvest treatments.

The strongest correlation between sugars were found between fructose and glucose (0.81) when compared to that between glucose and sucrose (0.77) and between fructose and sucrose (0.66) (Table 1).

### 3.5 Real-time respiration rate

**Fig. 7:**
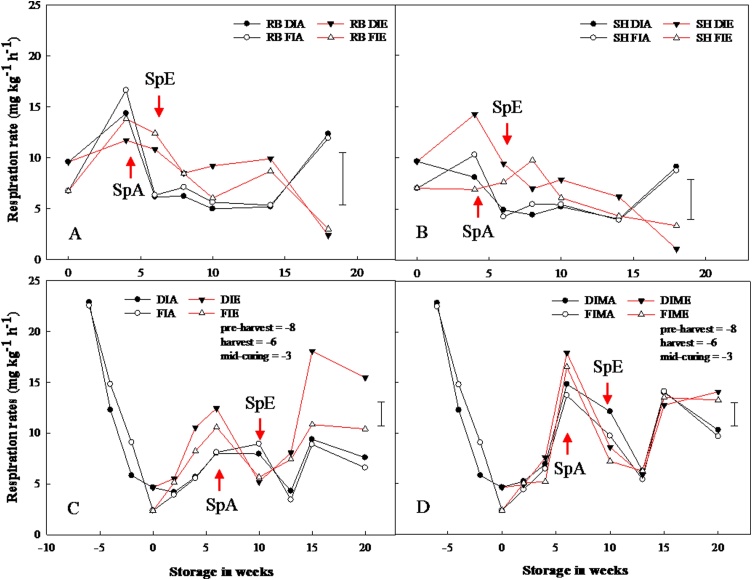
Real-time respiration rates as CO_2_ production (mg kg^–1^ h^–1^) of stored onion bulbs. Onion bulbs of cultivar ‘Red Baron’ (RB) and ‘Sherpa’ (SH) were grown in 2015 (A and B) and ‘Sherpa’ in 2016 (C and D) under full irrigation (FI) or deficit irrigation (DI); where FI amounted to 100% replenishment of crop evapotranspiration (ET_c_) and DI was ET_c_ replenishment at 50%. Bulbs were harvested at full maturity (100% fall-down) and cured under glass for six weeks for both years. Post-curing, bulbs were treated with or without 1-MCP at 1 μL L^−1^ for 24 h before storage; and then, stored at 1 °C under continuous ethylene supplementation at 10 μL L^−1^ or air. DIA and FIA are DI and FI bulbs in air, DIE and FIE are DI and FI bulbs stored under ethylene, DIMA and FIMA are DI and FI bulbs treated with 1-MCP and stored in air and DIME and FIME are DI and FI bulbs treated with 1-MCP and stored under ethylene. SpA and SpE are sprout emergences for bulbs stored in air and ethylene, respectively. LSD bar at 95% confidence shown.

In 2015, real-time respiration rates (RR) measured after curing prior to storage were 9.58 and 6.73 mg kg^−1^ h^−1^ of CO_2_ for ‘Red Baron and 9.64 and 7.03 mg kg^−1^ h^−1^ of CO_2_ for ‘Sherpa’; for DI and FI bulbs, respectively (Fig. 7 A and B). At the end of curing, RR was not significantly different between pre-harvest treatments within cultivars. After four weeks of storage, mean RR increased by at least 50 and 25% for ‘Red Baron’ and ‘Sherpa’ bulbs, respectively. Overall, the mean RR during storage was significantly higher in cv. ‘Red Baron’ (by 25%) when compared to ‘Sherpa’ for the 2015 experiment.

To understand the impact of curing on RR, during the 2016 experiment, RR was measured for onion bulbs two weeks prior to harvest (pre-harvest), at harvest, at mid-curing, and after curing. RR declined continuously from 22.8 mg kg^−1^ h^−1^ of CO_2_ at pre-harvest to 4.6 mg kg^−1^ h^−1^ of CO_2_ at the end of curing (i.e. pre-harvest > harvest > mid-curing > post-curing). Although RR declined 6-fold from pre-harvest to end of curing, there was no significant differences in RR between DI and FI bulbs (Fig. 7 C and D). During cold storage, RR increased steadily until dormancy-break at week 6 (7.9–18.0 mg kg^−1^ h^−1^ of CO_2_), when the RR for 1-MCP-treated bulbs were twice as high when compared to the untreated bulbs stored under ethylene or air. The RR at dormancy-break were: 1-MCP treated bulbs under ethylene > 1-MCP treated bulbs stored in air > bulbs stored under ethylene > bulbs stored in air. Thereafter, RR declined until week 13, after which the RR for bulbs stored under ethylene became significantly higher in DI compared to FI bulbs.

### 3.6 Dry matter content

Mean dry matter content (DMC) for DI and FI onion bulbs across all postharvest treatments and time points was 127.3 and 129.1 g kg^−1^ FW, respectively. There were no significant differences in DMC between DI and FI at pre-harvest, harvest, and throughout the curing period. DMC fluctuated during storage, and after 20 weeks DW was 17% higher in the FI compared to DI bulbs stored in air, while there was no significant difference between DI and FI bulbs stored under ethylene.

## 4 Discussion

Evapotranspiration, soil moisture content and curing weight-loss were significantly higher in the fully irrigated compared to deficit irrigated plants

Onion plants grown under deficit irrigation (DI) recorded a lower ET_c_ when compared to plants under full irrigation (FI) for both years. This showed that the pre-harvest treatment in this study successfully created differential transpiration between FI and DI plants, as was previously reported by Begum et al. (1993); thus validating the use of resulting bulbs in the postharvest studies reported herein.

### 4.1 Fructans redistribution prior to sprouting may predict dormancy-break

Fructans of higher DP initially present across all bulb sections at pre-storage could only be detected in the baseplate prior to sprouting. Fructans are polymers of fructose and the major reserve carbohydrates of onion. The concentration of fructans was reported to be highest at harvest, but decreased continuously to a minimum at dormancy-break and sprouting during storage (Suzuki and Cutcliffe, 1989; Jaime et al., 2001). The data herein showed that whilst this is true for the fructans content in the top and bottom sections, the opposite was true for the fructan content in the baseplate region; where the fructans content increased continuously until sprout emergence after which it declined (Fig. 2). However, previous studies on onion fructans sampled either the whole bulb (Suzuki and Cutcliffe, 1989), equatorial sections (Darbyshire and Henry, 1978) or the inner scales (Jaime et al., 2001) of onion bulbs. This study here represents the first investigation of fructans content in baseplate of onion bulbs. The reasons behind this accumulation of fructans in the baseplate prior to sprouting is unclear; though they quickly declined after sprout emergence. Notably, neither ethylene nor 1-MCP affected this decline. Notwithstanding this, specific accumulation of fructans in the baseplate prior to sprouting may have been to ensure adequate reserve energy was available for the successful initiation of sprouting whilst also offering osmo-protection to the meristematic tissues.

Fructans vary in their degree of polymerisation (DP) and are known to possess some osmo-regulation activities (Asega and Machado De Carvalho, 2004; Garcia et al., 2011). The higher accumulation of kestose (a DP3 fructan) content in the bottom section of DI and in the baseplates of FI bulbs between pre-harvest and mid-curing shown here (Fig. 3) was thought to be in response to water-stress. More so, these differences occurred in the sections closer to the roots where water availability differed between DI and FI plants. Interestingly, the differences in kestose content had disappeared at the end of curing, which suggested that this accumulation of kestose was not related to dormancy. In addition, the higher DP fructans content did not vary according to the imposed irrigation regime. Since, kestose is the first and last product of fructan biosynthesis from sugars and fructan catabolism to sugars, respectively, their biosynthesis and catabolism may be central to osmoregulation in onion plants under water-stress. This is supported by the equal and strong positive correlations of 0.83 shown by kestose to sucrose and nystose (Table 1). Nevertheless, the differences in kestose content between pre-harvest treatments were no longer significant in cured bulbs prior to storage. Overall, correlations between fructans diminished with increasing differences in DP as shown in Table 1. Fructans accumulation had previously been reported in transgenic tobacco leaves and roots (Pilon-Smits et al., 1995a; Li et al., 2007), rice leaves (Kawakami et al., 2008) and sugar beet roots and shoots (Pilon-Smits et al., 1999), grown under drought-stress. While these allude to a role for kestose content prior to end of curing, it contrasts with data on total fructan content. This then suggests that fructans may be recycled in relation to the plants’ responses to certain stimuli (in this case drought stress) without affecting total fructan content. Therefore, the accumulation of fructans in response to drought-stress may be typical of fructan type, plant tissues and species.

A positive relationship between onion bulb higher DP fructans content and delayed sprouting has been previously reported; such that long storing onion bulbs are known to typically have higher accumulation of higher DP fructans while poorer storing onion bulbs tend to accumulate low DP fructans (Jaime et al., 2001). While this suggests that the accumulation of higher DP fructans may extend dormancy, the data presented herein is at odds with this understanding. ‘Red Baron’ had a higher fructans content, including those of higher DP, when compared to ‘Sherpa’, however, ‘Red Baron’ sprouted two weeks earlier. This suggests that the accumulation of higher DP fructans in the whole bulb alone is not sufficient to predict dormancy-break or length of storage of onion bulbs.

Fructans of DP3-7 were present across all sections of the onion bulb before harvest, while fructans of DP8 also present in the top and bottom wedges, was conspicuously absent from the baseplate. Previous authors (Suzuki and Cutcliffe, 1989; Jaime et al., 2001; Benkeblia et al., 2006; Chope et al., 2012) reported that the concentration of fructans declined to a minimum, which coincided with dormancy-break and sprout emergence. Understandably, dormancy-break and sprout emergence had not been clearly differentiated in previous onion studies; thus, they are used interchangeably. Dormancy-break is the release of meristematic tissues for regrowth – at which point there might not be any physical signs of sprouting; while sprout emergence is when there is a physical appearance of sprout. Furthermore, considering the bulb sections sampled, it becomes apparent why previous studies reported a decline in fructans content at dormancy-break and sprout emergence. The data herein has shown for the first time that: (1) fructans of DP7 and 8 became conspicuously absent from the top and bottom wedges two and six weeks prior to sprout emergence (for bulbs stored in air and ethylene, respectively); and (2) the decline of fructans from the top and bottom wedges coincided with the accumulation of higher DP in the baseplate. This phenomenon suggests a top-to-bottom remobilisation of higher DP fructans. Notably, even though ethylene and 1-MCP treatments delayed and suppressed sprout growth, neither treatments affected the decline of these fructans, thereby suggesting that ethylene-related sprout suppression, while effective in the regulation of eco-dormancy, may not be involved in mediating endo-dormancy. Nevertheless, it was unclear if this redistribution of fructans of DP7 and 8 resulted from or was caused by dormancy-break.

Total sugar content declined across all sections at dormancy-break irrespective of pre- or postharvest treatments. Sugars are substrates for fructans biosynthesis where the first step in the fructans biosynthesis pathway involves the transfer of the fructosyl group from one sucrose to another sucrose creating kestose, a DP3 fructan. This first step is catalysed by sucrose:sucrose 1-fructosyltranferase (1-SST); while the elongation of the fructan chain (increase in degree of polymerisation) is catalysed by fructan:fructan 6(G) fructosyltranferase (6G-FFT) (Vijn et al., 1998; Lüscher et al., 2000). Catabolism of fructans into its individual sugar components is by hydrolysis, which releases the fructose moiety; catalysed by fructan 1-exohydrolase (1-FEH) (Van Den Ende et al., 2003). The total sugar content data herein showed an increase in the first two weeks of storage across all sections; which coincided with reduced real-time respiration rate (RR) (Fig. 7). Thereafter, the total sugar content declined prior to sprout emergence (Fig. 5) at which point there was an increase in respiration rate. Individual sugars (*viz*. fructose, glucose and sucrose) content varied during storage and more so at sprout emergence. Pre-harvest fructose content increased continuously during storage, irrespective of a steep decline in RR from pre-harvest to end of curing; and this coincided with the decline in fructans as reported herein and elsewhere (Suzuki and Cutcliffe, 1989; Jaime et al., 2001; Benkeblia et al., 2006). Furthermore, there was a spike in the concentration of fructose and sucrose across all sections for bulbs stored in ethylene, which was more obvious for 1-MCP-treated bulbs stored in ethylene. The reason behind this transient increase is unclear, since there was also a spike in the RR for these bulbs. However, this finding is also supported by Chope et al. (2006a) who previously reported an increased accumulation of sugars in 1-MCP treated onion bulbs. Dissimilar to fructose, glucose content declined by a half after four weeks of storage irrespective of pre- or postharvest treatments. Following this decline, sprouts were recorded two and six weeks later for bulbs stored in air and ethylene, respectively, and coincided with the disappearance of DP7 and 8 fructans from the top and bottom wedges and concomitant accumulation in the baseplate. The observation suggests that glucose may be the preferred source of energy at dormancy-break. Interestingly, neither pre- nor postharvest treatments influenced the decline in glucose prior to sprouting; suggesting that neither ethylene nor 1-MCP is involved in endodormancy-break but instead in eco-dormancy (sprout suppression).

Three classes of dormancy exist *viz*. endo-dormancy, para-dormancy and eco-dormancy; where endo-dormancy is time-dependent, irrespective of the environmental condition, para-dormancy is dependent on the transfer of biochemical compounds, while eco-dormancy is dependent on environmental conditions (Campbell, 2006). Endo-dormancy is thought to occur at around bulb initiation, when the growth of all meristematic tissues is arrested (Terry et al., 2015). Notably, significant changes in sugar concentration occurred only after sprout emergence.

## 5 Conclusions

Deficit irrigation had no effect on sprout emergence, or the accumulation or distribution of total fructans content within the onion bulbs under cold storage. Therefore, the accumulation of fructans and the degrees of polymerisation thereof in onion bulbs may be genetically driven rather than being dependent on pre-harvest irrigation regimes. Ethylene as an independent factor delayed sprout emergence and when combined with 1-MCP produced the shortest sprouts as reported by (Cools et al., 2011); however, neither significantly influenced the fructans content within the onion bulbs in relation to dormancy-break. This suggests that the mechanism by which ethylene and 1-MCP reduce sprouting may not be through fructans remobilisation. Prior to sprout emergence and regardless of pre- or postharvest treatments, fructans of DP7 and 8 were redistributed from the top and bottom sections of the bulb to the baseplate - in a para-dormancy-like remobilisation. It is unclear whether this redistribution resulted from, or was caused by dormancy-break; however, it occurred prior to sprout emergence. As such, although ethylene and 1-MCP treatments influenced eco-dormancy through delaying sprouting, neither seemed to affect endo-dormancy. Furthermore, neither the interactions between pre-harvest irrigation, postharvest ethylene nor 1-MCP treatments significantly influenced the accumulation and redistribution of fructans within the onion bulb in relation to dormancy-break and sprouting. Given these findings, the redistribution of fructans prior to sprouting could serve as a potential marker to predict the transition from endo-dormancy.

## Declaration of interests

None.

## Figures and Tables

**Fig. A1:**
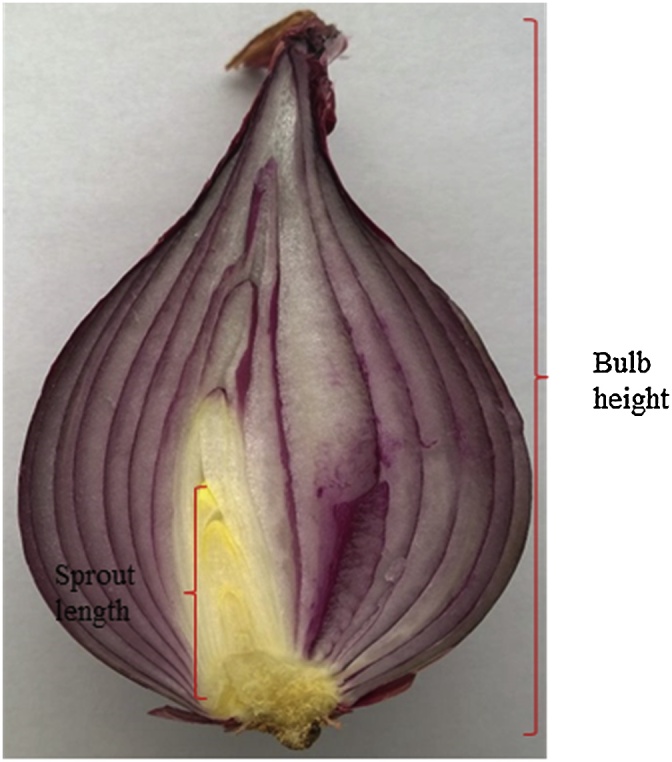
Depiction of sprout assessment.

**Fig. B1:**
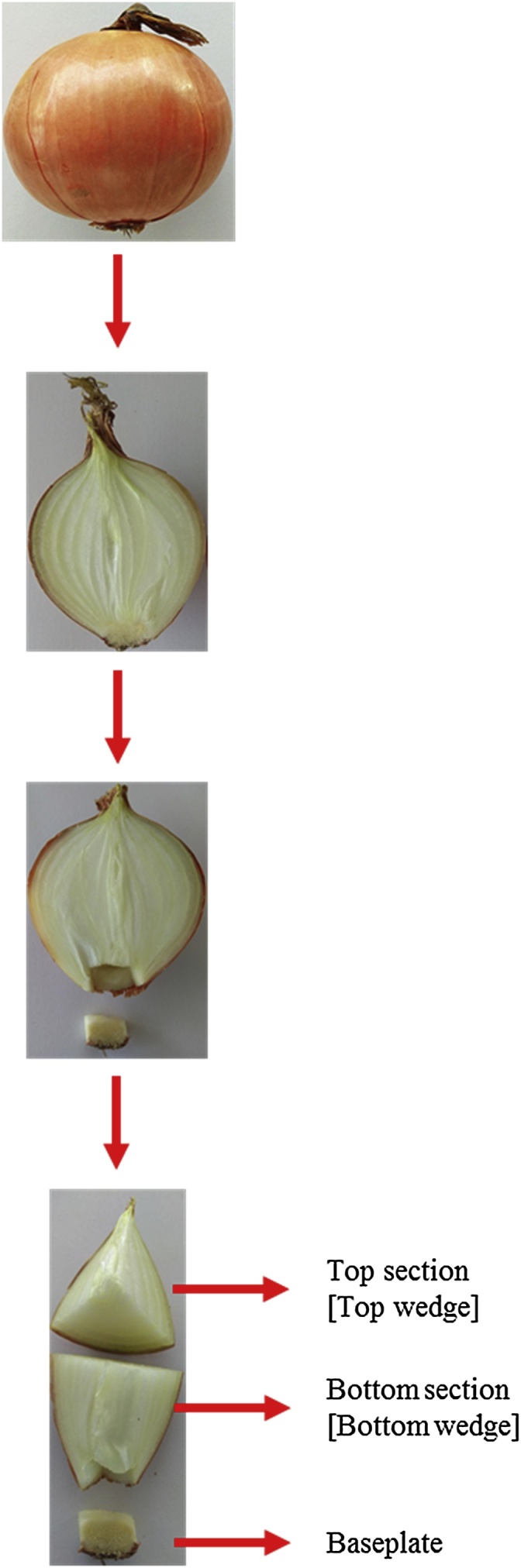
Onion bulb cut into top wedge, bottom wedge and baseplate.

**Fig. C1:**
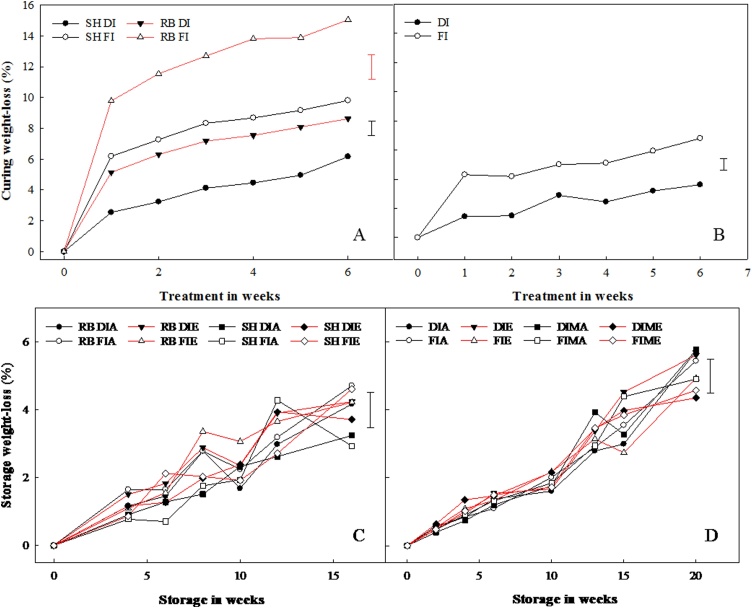
Postharvest curing weight-loss (A and B) and storage weight losses (C and D) in stored onion bulbs. Onion bulbs of cultivar ‘Red Baron’ (RB) and ‘Sherpa’ (SH) were grown in 2015 (A and C) and 2016 (B and D) under full irrigation (FI) or deficit irrigation (DI); where FI amounted to 100% replenishment of crop evapotranspiration (ET_c_) and DI was ET_c_ replenishment at 50%. Bulbs were harvested at full maturity (100% fall-down) and cured under glass for six weeks for both years. Post-curing, bulbs were either treated with 1-MCP at 1 μL L^−1^ for 24 h or untreated before storage; and then, stored at 1 °C under continuous ethylene supplementation at 10 μL L^-1^ or air. DIA and FIA are DI and FI bulbs in air, DIE and FIE are DI and FI bulbs stored under ethylene, DIMA and FIMA are DI and FI bulbs treated with 1-MCP and stored in air and DIME and FIME are DI and FI bulbs treated with 1-MCP and stored under ethylene. LSD bar at 95% confidence shown.

**Fig. D1:**
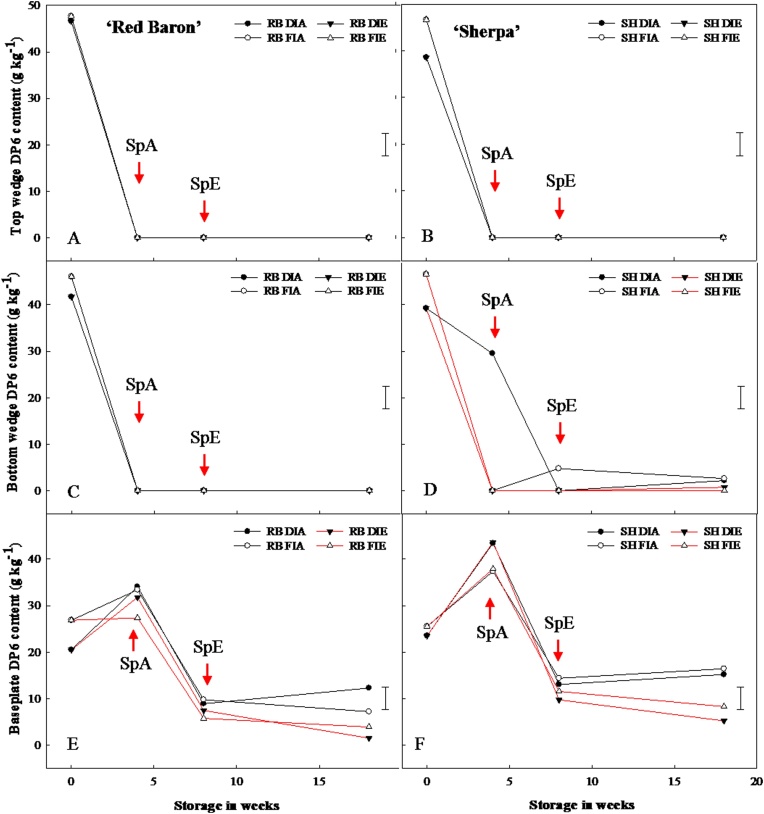
Pre- and postharvest fructans DP6 content in stored onion bulbs. DP6 fructans contents per dry weight for the top wedge (A and B), bottom wedge (C and D) and baseplate (E and F) of onion bulbs of cultivar ‘Sherpa’ grown in 2015 under full irrigation (FI) or deficit irrigation (DI); where FI amounted to 100% replenishment of crop evapotranspiration (ET_c_) and DI was ET_c_ replenishment at 50%. Bulbs were harvested at full maturity (100% fall-down) and cured under glass for six weeks for both years. Post-curing, bulbs were stored at 1 °C under continuous ethylene supplementation at 10 μL L^−1^ or air. DIA and FIA are DI and FI bulbs stored in air and DIE and FIE are DI and FI bulbs stored under ethylene. SpA and SpE are sprout emergences for bulbs stored in air and ethylene, respectively. LSD bar at 95% confidence shown.

## Appendix A Sprout assessment

Fig. A1

## Appendix C Curing weight-loss and Storage weight-loss

Fig. C1

## Appendix D DP6 fructans in the top, bottom and baseplate (2015)

Fig. D1
